# Supplementary material for: All of gene expression (AOE): An integrated index for public gene expression databases
Source: PLoS One. 2020 Jan 24;15(1):e0227076. doi: 10.1371/journal.pone.0227076 (PMC6980531; doi:10.1371/journal.pone.0227076)
Supplement: Attachment — Submitted filename: 191207rebuttal.pdf [file pone.0227076.s001.pdf]

# Response to Reviewer #1

> 1. Regarding the usage statistics:
> "From the fact that most visits were under 30seconds, it seems that users accessed AOE web server in their web browser with an instant query with specific keywords."
> - It could also be first time visitors who do not spend much time on the page.
> - Are there additional metrics available to identify such users?

Thanks for clarification. We are using awstats (https://awstats.sourceforge.io) to analyze httpd log, and there is a statistics for 'visits duration'.
While the analyzed page can be browsed by months, percentages of 'visit duration' under 30s are around 66%. Below is an example for the latest statistics for that(in Oct 2019).
In other words, two thirds of web access are below 30s.
Thus, we changed the description about usage statistics in the Results section.
"The fact that two-thirds of visits were under 30seconds indicates that users accessed the AOE web server in their web browser with an instant query with specific keywords."

**Visits duration**

| Number of visits: 1,308 - Average: 932 s | Number of visits | Percent |
|---|---|---|
| 0s-30s | 858 | 65.5 % |
| 30s-2mn | 19 | 1.4 % |
| 2mn-5mn | 10 | 0.7 % |
| 5mn-15mn | 14 | 1 % |
| 15mn-30mn | 75 | 5.7 % |
| 30mn-1h | 89 | 6.8 % |
| 1h+ | 235 | 17.9 % |
| Unknown | 8 | 0.6 % |

> 2. Phrasing of sentences through out the paper:
> "AE once had imported data from GEO, but stopped importing data in 2017"
> - could be rephrased to "AE had once imported data from GEO, but stopped doing so in 2017."
> "Integration of these public gene expression databases is required"
> - could be rephrased to "There is a need for integration of these public gene expression databases."
> "AOE was originally begun as an index for the ArrayExpress (AE) database maintained at EBI"
> - could be rephrased to "AOE originally began as an index…"

Thank your very much for your editing.
All issues were rephrased.

# Response to Reviewer #2

> Reviewer #2: Dear author, thank you for addressing my comments. I think the paper will benefit from some minor editing. Nothing serious, but I am sure a native speaker can be helpful to get it up to PLoS standards. Maybe the editor can recommend someone.

Thank you very much for your comment.
The manuscript was re-edited by another editor, and English was very much improved.
I attached a PDF file of 'CERTIFICATE OF ENGLISH EDITING'.
